# Multiple Opportunistic Infections as the Initial Presentation of Human Immunodeficiency Virus (HIV)/Acquired Immunodeficiency Syndrome (AIDS) in an Ecuadorian Immigrant

**DOI:** 10.7759/cureus.106368

**Published:** 2026-04-03

**Authors:** Uma D Alappan, Trevan Klug, Jessica Magid-Bernstein, Anita Huttner, Joseph Vinetz

**Affiliations:** 1 Internal Medicine, Yale School of Medicine, New Haven, USA; 2 Pathology, Yale School of Medicine, New Haven, USA; 3 Neurology, Yale School of Medicine, New Haven, USA; 4 Infectious Disease, Yale School of Medicine, New Haven, USA

**Keywords:** aids, immigrant health, neurology, opportunistic infection, pathology

## Abstract

Advanced human immunodeficiency virus (HIV)/acquired immunodeficiency syndrome (AIDS) continues to occur in those with social, educational, and cultural barriers to accessing healthcare. A 27-year-old Ecuadorian man presented with vomiting, epigastric pain, fever, weakness, and weight loss. He had advanced AIDS, with opportunistic infections including disseminated histoplasmosis, cerebral toxoplasmosis, and JC virus brain infection. Despite prior repeated contact with local, US-based healthcare, sexual history and HIV screening were never performed. Missed opportunities to identify HIV infection led to potentially preventable opportunistic infections, despite our current era of widely available, effective antiretroviral therapy.

## Introduction

Delayed diagnosis of human immunodeficiency virus (HIV) continues to occur and places patients at risk for severe opportunistic infections. In the United States in 2023, among 38,793 people diagnosed with HIV, 21.6% received stage 3 (acquired immunodeficiency syndrome (AIDS)) classification at the time of HIV diagnosis [1]. Ecuador and other Latin American countries have a high burden of endemic mycoses and other invasive fungal infections in both healthy individuals and hosts at increased risk for infection [2]. Progressive disseminated histoplasmosis has been considered an AIDS-defining illness in South America since 1987, though underdiagnosis is frequent [3]. Cases of histoplasmosis infection in Ecuador were first described in 1999 in immunocompromised individuals and in 2002 for patients with AIDS [2,4]. Despite Centers for Disease Control and Prevention (CDC) recommendations for routine HIV screening, patients, including immigrants, the uninsured, or those with paucity of contact with the healthcare system, often fall through the gaps [5,6]. We describe a case of multiple opportunistic infections as the initial presentation of HIV/AIDS in Connecticut in a young immigrant from Ecuador despite prior contact with the healthcare system.

## Case presentation

**Figure 1:**
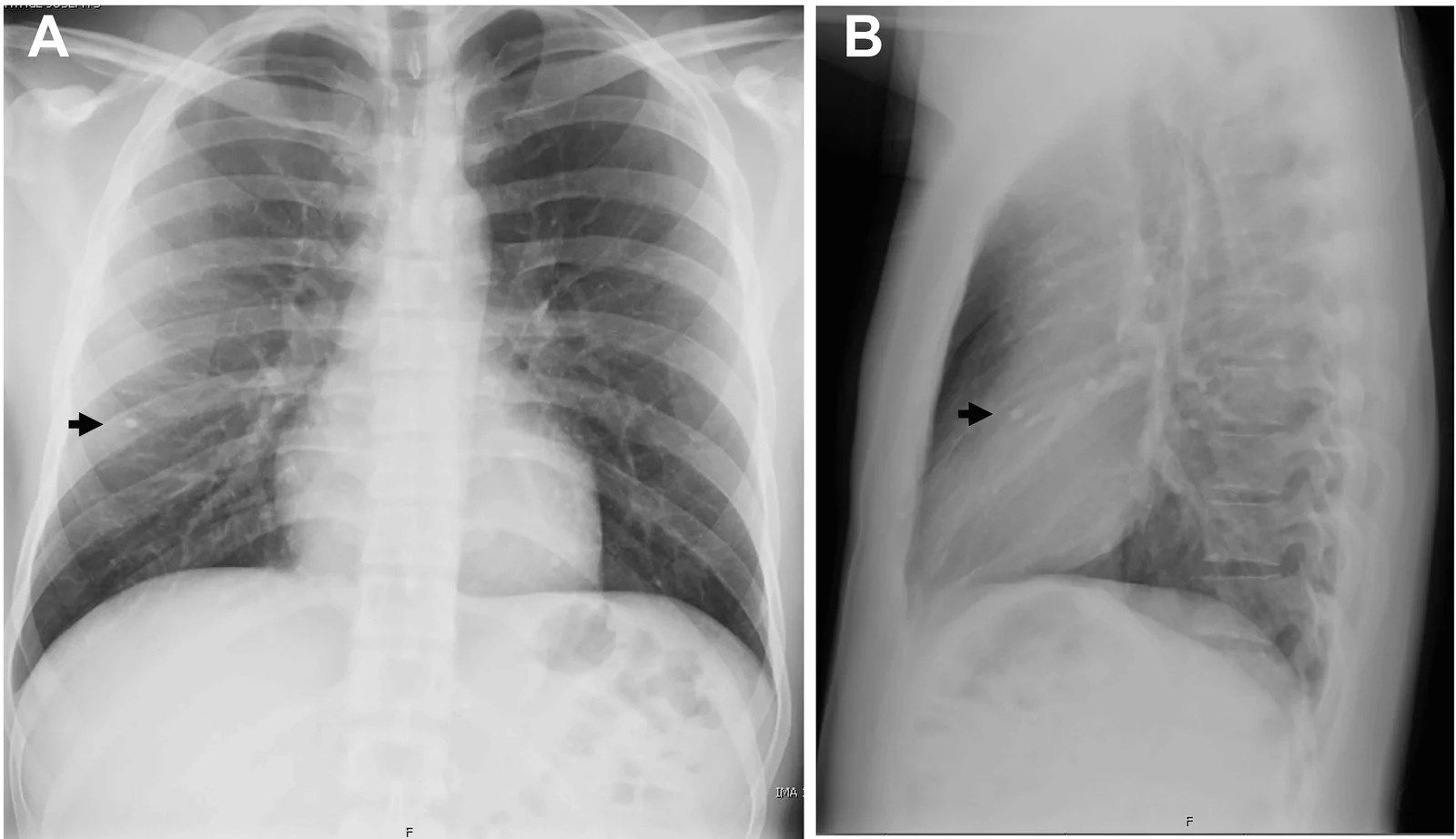
PA and lateral chest X-ray from one year before presentation A single calcified granuloma (arrow) is present in the right middle lobe, and probable hilar lymph node calcifications are seen, without acute abnormality. PA: posterior-anterior

A 27-year-old man, who had immigrated from Ecuador in 2023, presented with a two-day history of non-bloody vomiting, coughing, epigastric pain, night sweats, and 17 lb. unintentional weight loss over the previous three weeks. In the previous year, he had been seen in an emergency department (ED) for fever, pharyngitis, abdominal pain, and myalgia. Chest X-ray showed a small, calcified granuloma in the right middle lobe (Figure 1).

**Figure 2:**
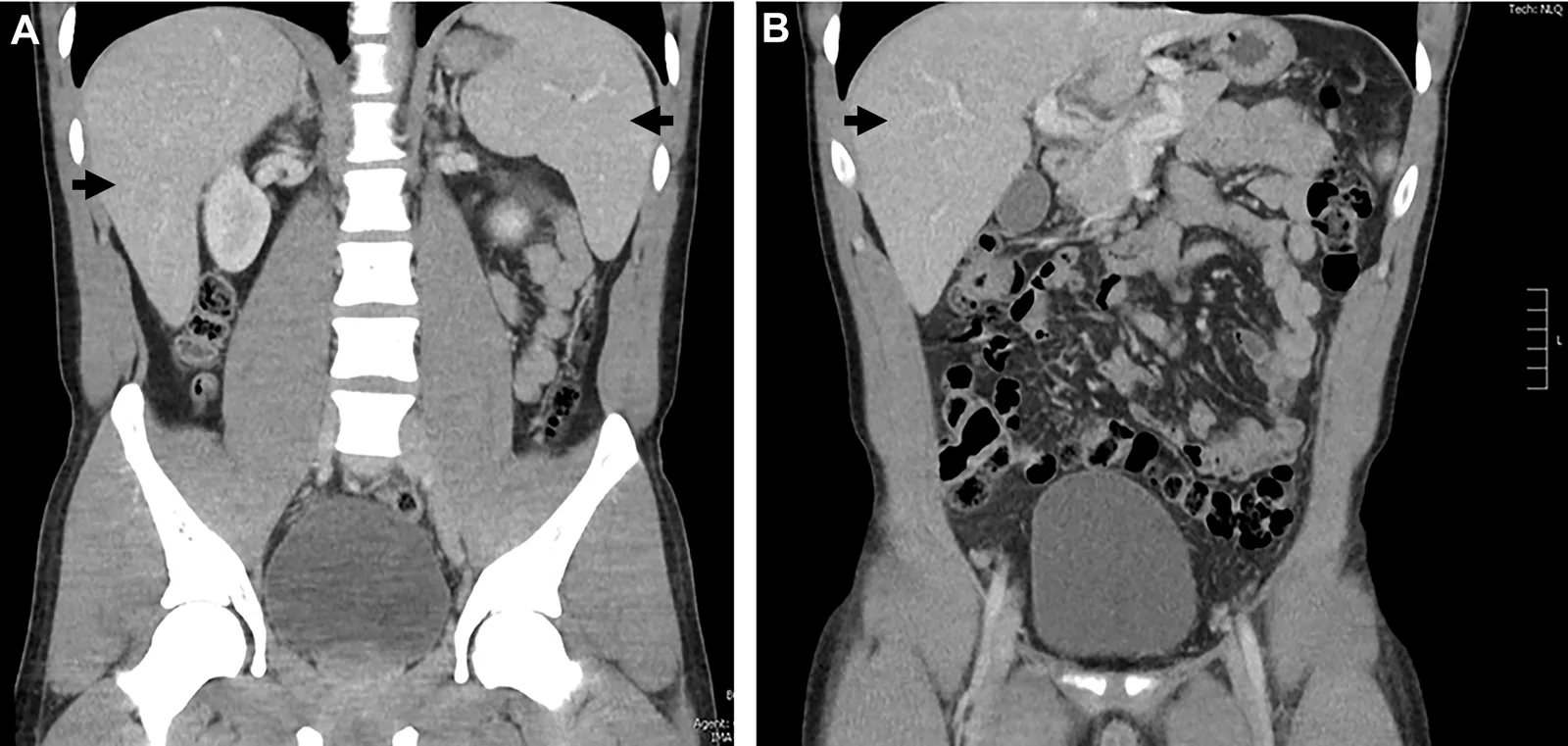
CT of the abdomen and pelvis with intravenous contrast No abnormality was seen, including no calcifications in the liver or spleen. Two cuts are presented ((A) anterior; (B) posterior). Rightward arrows indicate the liver; leftward arrow indicates the spleen. CT: computed tomography

Computed tomography (CT) imaging of the abdomen and pelvis was unrevealing (Figure 2).

Despite a positive heterophile (Monospot) test five years before, positive Epstein-Barr virus (EBV) serological testing (positive for IgM antibodies to VCA antigen, positive for IgG antibodies to VCA and EBNA-1 antigens) led to a discharge diagnosis of mononucleosis. Three months later, he was evaluated for bloody diarrhea and abdominal pain, but he was not able to have standard testing done, presumably for financial and language reasons. No vaccination or sexual history was obtained, nor was routine HIV screening done.

Seven months later, the patient presented to our hospital in Connecticut with fever, tachycardia, and abdominal pain with generalized tenderness to palpation without peritoneal signs. Neurological examination was normal. Workup revealed pancytopenia (white blood count: 3.3×1,000/uL (reference range: 4,000-11,000/uL); hemoglobin: 11.5 g/dL (reference range: 13.2-17.1 g/dL); platelet count: 46,000/uL (reference range: 150-400/uL)). Pancytopenia led to HIV testing which showed HIV-1 seropositivity, a viral load of 504,000 copies/mL (normal: undetectable), and a CD4+ T-cell count of 8×1,000/uL (reference range: 466-1,608/uL). Testing for the hepatitis B virus and hepatitis C infection was negative. HIV-1 genotyping showed that his infecting virus was HIV-1 subtype B. No resistant mutations to nucleoside/nucleotide reverse transcriptase inhibitors, non-nucleoside reverse transcriptase inhibitors, or protease inhibitors were detected. Possible or emerging resistance mutations were detected in reverse transcriptase (T69T/A, K101Q, V179I) and protease inhibitor resistance (L63P, H69Q/H, V77I/V, V82I) genes.

The patient grew up on a farm in Ecuador, had no pets, no known tuberculosis exposure, and no known positive skin test for tuberculosis exposure, and had traveled through the American southwest prior to arriving in New England. He had alcohol use disorder but denied IV drug use and tobacco/smoking. He reported having sex with women since he was 17 years old and denied having had sex with men or with sex workers of any gender. He did report unprotected sex while inebriated. He had never been tested for sexually transmitted infections.

**Figure 3:**
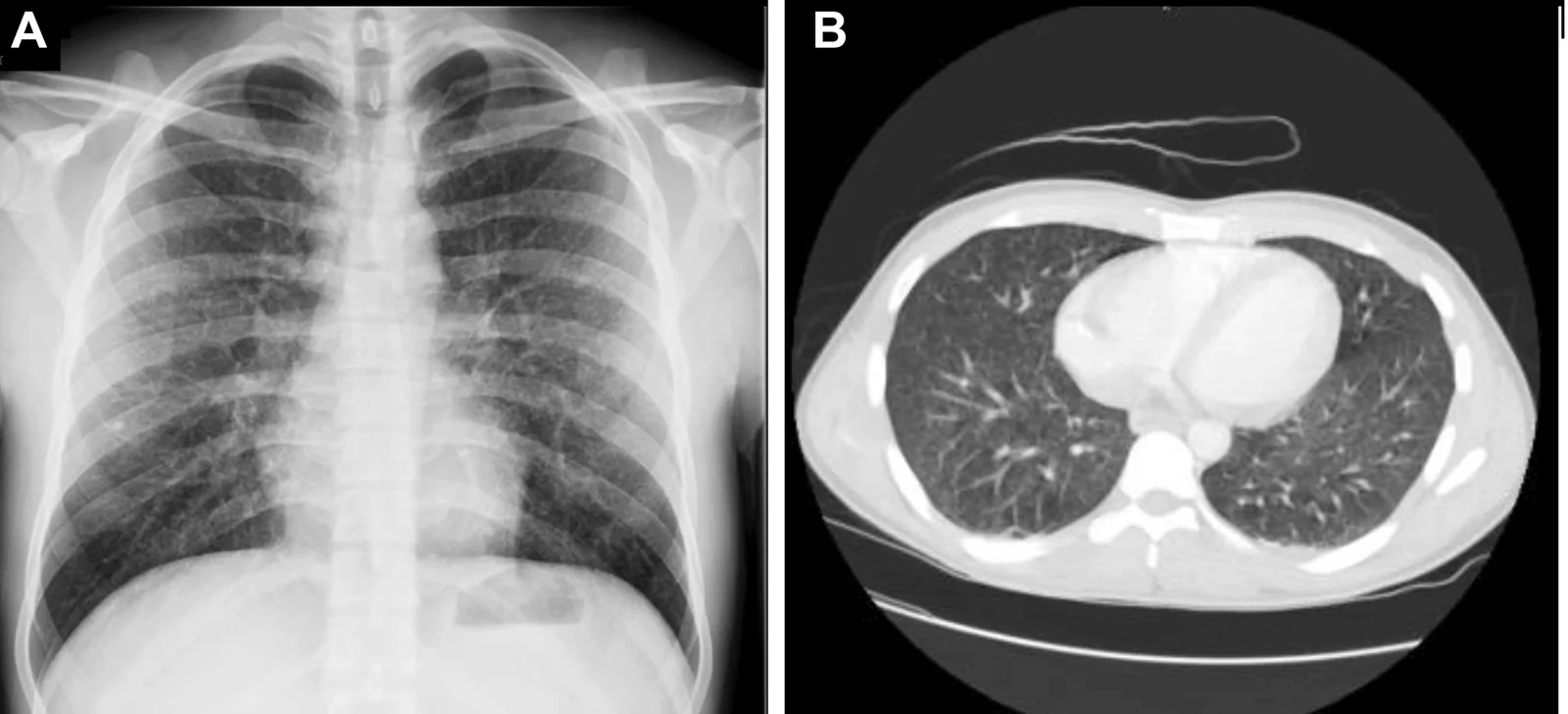
(A) PA chest X-ray and (B) CT imaging of the chest at the time of clinical presentation (A) PA chest X-ray at the time of the current presentation. (B) CT of the chest. Both images show diffuse, bilateral miliary distribution of micronodules in the lungs, suggesting miliary tuberculosis or other atypical infections such as histoplasmosis. PA: posterior-anterior; CT: computed tomography

Further workup included a CT of the chest with IV contrast, which showed diffuse, bilateral lung micronodules concerning for miliary tuberculosis or atypical infection (Figure 3).

**Figure 4:**
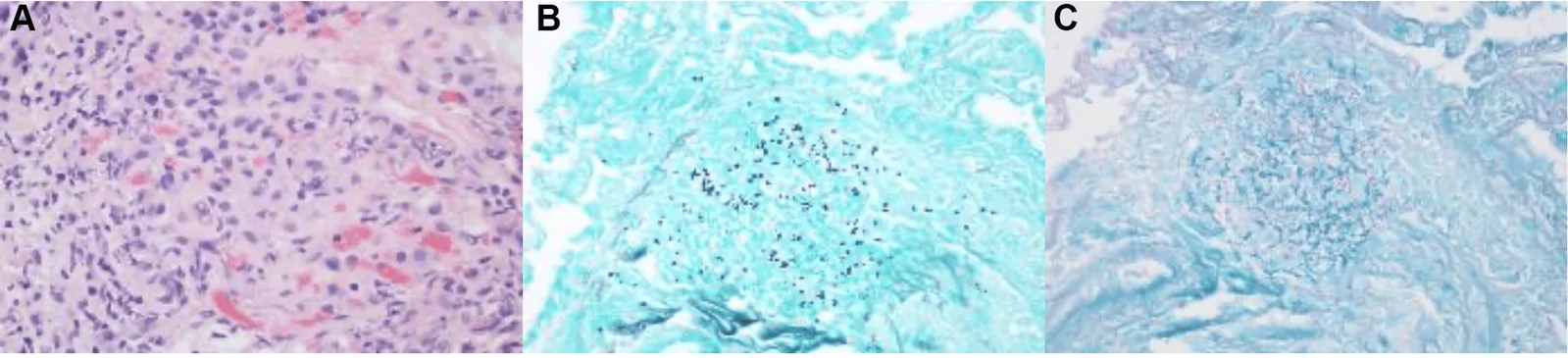
Histopathologic analysis of transbronchial biopsy (A) H&E staining demonstrating acute and chronic inflammation and poorly formed granulomas. (B) GMS strongly staining small yeast forms with narrow based budding. (C) PAS-fungus weakly positively staining numerous small yeast forms with narrow based budding. Magnification, 40×. H&E: hematoxylin and eosin; GMS: Grocott-Gömöri's methenamine silver; PAS: periodic acid-Schiff

Additional workup included bronchioalveolar lavage (BAL) and transbronchial biopsy. BAL grew 700 CFU/mL of *Histoplasma* *capsulatum* on fungal culture. Serum assay for beta-D-glucan (Fungitell) was positive (217 pg/mL; negative <60), and urine *H. capsulatum* antigen was positive. Lung biopsy obtained during fiberoptic, flexible bronchoscopy grew *H. capsulatum*, with negative mycobacterial, bacterial, and *Nocardia* and *Actinomyces* cultures. Histopathologic analysis of this lung tissue demonstrated organizing pneumonitis with acute and chronic inflammation and poorly formed granulomas. Grocott-Gömöri's methenamine silver (GMS) was strongly positively and periodic acid-Schiff (PAS) weakly positively staining for numerous small narrow budding yeast forms (Figure 4).

Tissue staining for acid-fast bacilli was negative (not shown). CT of the abdomen showed multiple retroperitoneal lymph nodes with splenomegaly and inflammation in the cecum and ascending colon. Blood polymerase chain reaction (PCR) for cytomegalovirus (CMV) showed a viral load of 2,900 IU/mL, raising concern in the context of the clinical radiographic findings for CMV vasculitis, colitis with associated appendicitis. No colonoscopy was performed. He was treated with liposomal amphotericin B for disseminated histoplasmosis and with oral valganciclovir for CMV infection and presumed CMV colitis/appendicitis.

**Figure 5:**
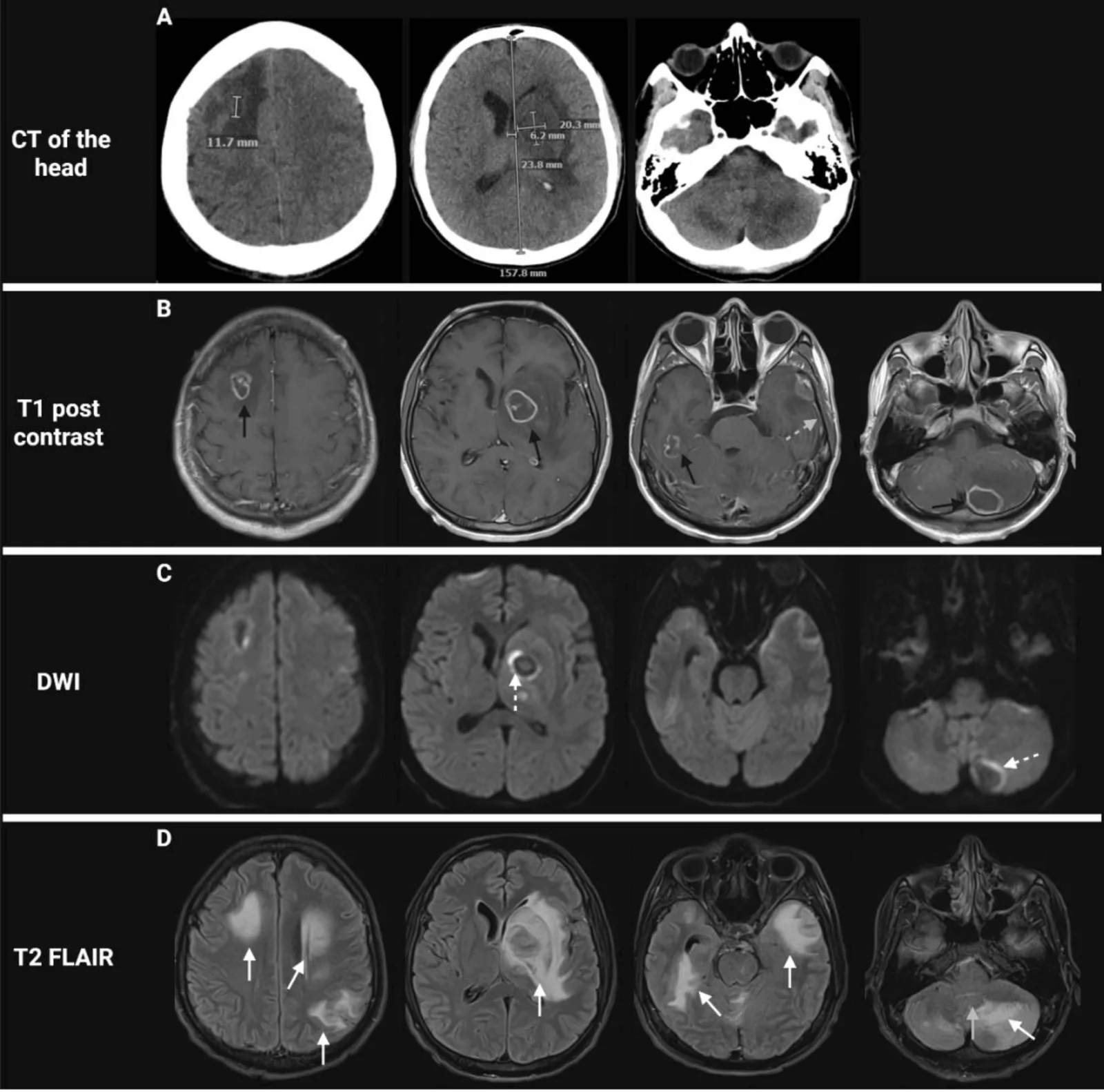
Cranial imaging showing multiple space-occupying lesions (A) CT of the head without IV contrast showing multiple supratentorial and infratentorial lesions with surrounding vasogenic edema, mass effect causing 6.2 mm midline shift, and fourth ventricular compression. (B-D) MRI of the brain with and without IV contrast demonstrated multiple ring-enhancing ((B) black arrows), peripherally diffusion-restricting ((C) white dashed arrows) supratentorial and infratentorial lesions with surrounding vasogenic edema ((D) white arrows) and mass effect on the fourth ventricle ((D) grey arrow). Some of these lesions were noted to have associated leptomeningeal enhancement ((B) grey dashed arrow). CT: computed tomography; MRI: magnetic resonance imaging; DWI: diffusion-weighted imaging; FLAIR: fluid-attenuated inversion recovery

Three weeks into admission, the patient became confused with expressive aphasia. Extensive brain imaging was performed (Figure 5).

CT of the head without IV contrast showed multiple supratentorial and infratentorial lesions with surrounding vasogenic edema, partial effacement of the fourth ventricle and near-complete effacement of the lateral ventricle, and regional mass effect with 6 mm midline shift (Figure 5A). He was empirically started on trimethoprim-sulfamethoxazole (TMP-SMZ) for toxoplasmosis and ceftriaxone, metronidazole, and vancomycin presumptively for bacterial brain abscesses. Vasopressin was started for potential central diabetes insipidus. Neurologic exam evolved to new right arm drift, decreased sensation to light touch and vibration in all extremities, and inability to protrude his tongue or smile. Initiation of steroids was deferred to avoid confounding lymphoma workup; antiretrovirals were deferred due to concern for initiating immune reconstitution inflammatory syndrome (IRIS). Magnetic resonance imaging (MRI) of the brain showed numerous, rim-enhancing, peripherally diffusion-restricting supratentorial and infratentorial lesions with vasogenic edema, mass effect on the fourth ventricle, and leptomeningeal enhancement (Figure 5B-5D). Lumbar puncture was deferred because of the risk of herniation. Further serological workup revealed positive *Toxoplasmosis gondii* IgG (>400 IU/mL), positive herpes simplex virus-1 (HSV-1) PCR of lip lesion, negative stool ova and parasite microscopic examination, and negative *Strongyloides* *stercoralis* antibody.

**Figure 6:**
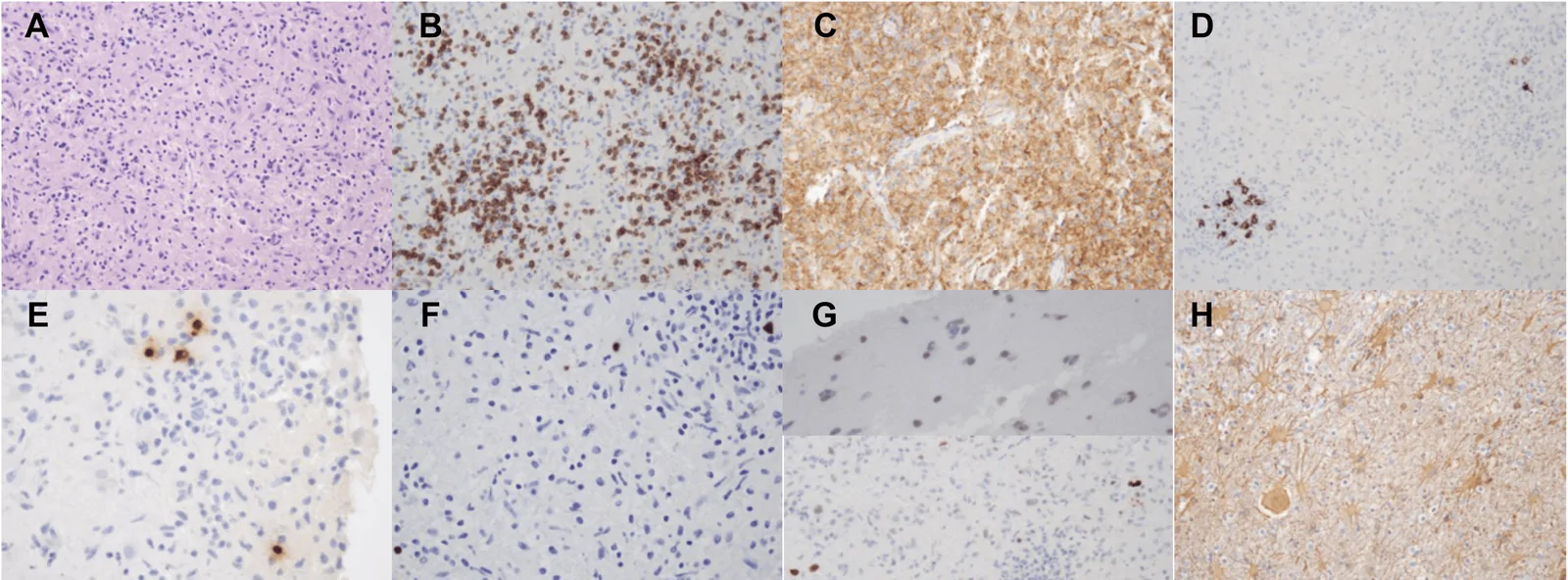
Histopathologic analysis of the right frontal brain lesion H&E. There is extensive inflammation with (A) dense perivascular intraparenchymal inflammatory infiltrate. The cells in the inflammation are a mixture of (B) CD3+ T lymphocytes, (C) CD163+ monocyte/macrophages, and (D) CD20-positive B lymphocytes. (E) *Toxoplasma gondii* immunostain demonstrated parasite cyst forms without tachyzoites. (F) EBV was present with occasional cells staining positive in the nucleus. (G) The JC polyoma virus was found in scattered oligodendroglial cells with slightly enlarged, "glassy"-appearing nuclei with positive immunoreactivity for SV40 (top) and (bottom). P53 is often used to identify JCV given the JCV T protein (which SV40 stains for) binds to and inactivates cellular P53. (H) GFAP staining showed "bizarre" giant astrocytes, consistent with PML. Magnification, 20×. H&E: hematoxylin and eosin; EBV: Epstein-Barr virus; JCV: JC virus; GFAP: glial fibrillary acidic protein; PML: progressive multifocal leukoencephalopathy

Histopathological and PCR analysis of the right frontal brain lesion biopsy (Figure 6) revealed *Toxoplasma gondii*, EBV, and JC virus infection, raising concern for progressive multifocal leukoencephalopathy (PML). *H. capsulatum* fungal forms were not identified in the brain biopsy.

Over the next several weeks, the patient stabilized clinically and radiographically on dexamethasone for his cerebral edema and PML, TMP-SMZ for toxoplasmosis, valganciclovir for CMV and HSV, and amphotericin B for histoplasmosis. Prior to discharge, he was started on antiretroviral therapy with raltegravir-emtricitabine-tenofovir disoproxil fumarate given favorable central nervous system (CNS) penetration with this regimen and itraconazole after the completion of a 14-day course of amphotericin B. He was scheduled for outpatient follow-up with the infectious disease clinic. On discharge, his neurological examination was normal.

## Discussion

This case highlights a systemic failure in the US healthcare system to detect HIV initially in a young immigrant to the United States with risk factors and two prior medical encounters with red flag symptoms, including lung granuloma, gastrointestinal symptoms, and unexplained EBV viremia. This patient had a lack of routine healthcare preventative screenings and no known vaccination history. Although the CDC recommends at least one-time HIV screening for all adults, this was never performed in our patient despite high-risk features [5]. It is possible that the development of opportunistic infections may have been prevented had diagnosis been obtained earlier and antiretroviral therapy been initiated during his initial contact with the healthcare system.

Several studies have shown high morbidity and mortality associated with disseminated histoplasmosis in patients living with HIV [7,8]. Living in coastal regions, living on farms with bat and bird droppings mixed in soil, working with poultry, and having a CD4+ T-cell count below 200/uL increases the risk of severe histoplasmosis in people with AIDS [2]. Our patient's story demonstrates the importance of investigating birthplace, migration history, and exposures in immigrant populations to the United States, as they may carry different risk factors than native-born US patients.

## Conclusions

Late HIV diagnosis continues to be a problem in the United States because of systemic barriers to what should be routine medical care. Consequently, this patient developed life-threatening opportunistic infections, reminiscent of the pre-antiretroviral era. Advanced AIDS-related immunosuppression and multiple infections resulted in diagnostic complexity, requiring invasive testing including brain biopsy, and ultimately required treatment with multiple anti-infectives. This case highlights the need for vigilance and early HIV testing in high-risk patients, especially those from endemic regions or with unexplained constitutional symptoms.
